# Increase in cotton yield through improved leaf physiological functioning under the soil condition of reduced chemical fertilization compensated by the enhanced organic liquid fertilization

**DOI:** 10.3389/fpls.2023.1225939

**Published:** 2023-08-23

**Authors:** Xiaojuan Shi, Xianzhe Hao, Aziz Khan, Nannan Li, Junhong Li, Feng Shi, Yu Tian, Jaya Nepal, Jun Wang, Honghai Luo

**Affiliations:** ^1^ Key Laboratory of Oasis Eco−Agriculture, Xinjiang Production and Construction Group, Shihezi University, Shihezi, Xinjiang, China; ^2^ Soil and Water Research Institute, Xinjiang Academy Agricultural and Reclamation Science, Shihezi, China; ^3^ Department of Soil, Water and Ecosystem Sciences, Indian River Research and Education Center, Institute of Food and Agricultural Sciences The University of Florida Institute of Food and Agricultural Sciences (UF/IFAS), Fort Pierce, FL, United States

**Keywords:** organic liquid fertilizer, photosynthetic performance, economic coefficient, seed yield, sustainable agriculture

## Abstract

**Introduction:**

Low agricultural nutrient input efficiency remains a significant impediment for crop production globally. To address this issue in cotton agroecosystems, there is a need to develop sustainable crop nutrient management strategies to achieve high crop yields. We hypothesized that organic liquid fertilizer (OF) combined with reduced chemical fertilizer (CF) would enhance cotton yield by improving leaf functioning and soil properties. However, the underlying mechanism and its related process is poorly understood.

**Methods:**

This study explored the effects of OF combined with reduced CF on cotton yield, physiology and soil properties. Treatments included a single application of CF (CF: N, P_2_O_5_ and K_2_O applied at 228, 131 and 95 kg ha^−1^) and combined applications of OF and CF (OF_0.6_−OF_1.4_) in the following ratios: OF_0.6_, OF+60% CF; OF_0.8_, OF+80% CF; OF_1.0_, OF+100% CF; OF_1.2_, OF+120% CF; OF_1.4_, OF+140% CF.

**Results and discussion:**

The result showed that compared with CF, OF_0.8_, OF_1.0_ and OF_1.2_ increased soil organic matter (SOM) content by 9.9%, 16.3% and 23.7%, respectively. Compared with CF, the OF_0.6_, OF_0.8_, OF_1.0_, and OF_1.2_ treatments increased leaf area (LA) by 10.6−26.1%, chlorophyll content (Chl content) by 6.8−39.6%, and the efficiency of photosystem II (PSII) light energy (Y(II)), electron transfer rate of PSII (ETR) and photochemical quenching (qP) by 3.6−26.3%, 4.7−15.3% and 4.3−9.8%, respectively. The OF_0.8_ treatment increased net photosynthetic rate (*P*_n_), stomatal conductance (*G*_s_) and transpiration rate (E) by 22.0%, 27.4% and 26.8%, respectively, resulting in higher seed cotton yield. The seed cotton yield and economic coefficient were positively correlated with *P*_n_, E, *G*_s_ and Y(II) from the full boll stage to the boll opening stage. In summary, the OF_0.8_ treatment can maintain a high SOM content and photosynthetic performance with reduced chemical fertilizer input without sacrificing yield. The integration of OF+80% CF (OF_0.8_) is a promising nutrient management strategy for highly efficient cotton production under mulch drip irrigation systems.

## 1 Introduction

Cotton is an economically important crop worldwide and represents a significant source of income for farmers in many countries. China, India, the United States, Pakistan, and Brazil are the top cotton-producing countries globally, accounting for more than 75% of the world’s cotton production (STATISTA, 2022). China ranks first globally, with 5.99 million tons of cotton production (Arshad et al., 2021). In 2020, Xinjiang’s cotton planting area was 25019 km^2^, and the total output was 5.2 million tons, accounting for 78.9% and 87.3% of the total cotton planting area and total output of China, respectively, and 8.82% and 22.3% of the total cotton planting area and total output of the world, respectively (National Bureau of Statistics, 2020). In the northwest inland cotton production region, the average amounts of applied N, P_2_O_5_ and K_2_O are 296 kg ha^−1^, 141 kg ha^−1^ and 87 kg ha^−1^ in the high-yield cotton region (7466 kg ha^−1^), while the average amounts of applied N, P_2_O_5_ and K_2_O are 278 kg ha^−1^, 116 kg ha^−1^ and 75 kg ha^−1^ in the low-yield cotton production region (5904 kg ha^−1^) (Li et al., 2019). The statistics above show that Xinjiang cotton production is characterized by a “high input, high yield and high risk” development status (Lou et al., 2021). Recently, there has been a shift toward more sustainable cotton production methods, such as organic and fair-trade cotton, in response to concerns about the environmental and social impacts of conventional cotton farming, especially the excessive use of conventional chemical inputs.

Excessive application of chemical fertilizer (CF) can increase cotton production costs and a decline in soil organic matter and soil fertility with no crop yield increment (Yan et al., 2007; Khan et al., 2017b). Therefore, it is necessary to develop efficient fertilizer management options to ensure high cotton yield with improved photosynthetic performance. The application of organic fertilizer can improve soil nutrients, soil physical and chemical properties, and crop yield while also promoting crop nutrient absorption (Agbede et al., 2010). Increasing organic fertilizer is beneficial for increasing crop yield and improving crop quality (Liu et al., 2017; Liang et al., 2021; Mousavi et al., 2022). The combined application of organic fertilizer and chemical fertilizer in an integrated manner can promote the absorption and utilization of chemical fertilizer (Kautz et al., 2004), enable efficient utilization of resources (Wright et al., 2004; Jagadamma et al., 2007). With the development of the chemical fertilizer industry, the amount of chemical fertilizer application in China has increased each year, while the proportion of organic fertilizer applied has decreased (Pan, 2014). The proportion of organic fertilizer relative to total fertilizer input decreased from 99.9% in 1949 to 37.4% in 1990 and 25.0% in 2003 (Xiao et al., 2017). Continuous, single and excessive applications of chemical fertilizer have resulted in low soil organic matter (SOM) contents and fertilizer utilization rates (Roelcke et al., 2004). However, most organic liquid fertilizers (OFs) are solid or biogas slurries with low efficiency that cannot meet the requirements of high crop yields or that contain heavy metals, resulting in environmental pollution (Yoder and Davis, 2020).

A new type of OF that is water-soluble and can activate soil nutrients has been shown to achieve efficient management of nutrient resources, resulting in high crop yields with less damage to the environment (Maintang et al., 2021). Coupling OF with CF has been shown to effectively improve the CF utilization rate, thereby significantly improving soil fertility and crop yield (Mooy et al., 2019; Puteri et al., 2021). OFs can boost soil texture, improve the crop growth environment, and enhance the accumulation of photosynthetic products, thus leading to an increase in cotton yield (Tian et al., 2014). Hence, understanding the effects of the application of OF combined with CF throughout the whole growth period on crop growth and yield formation is important to boost yield, particularly in arid regions. However, the interactions between OF with CF and the effect on cotton photosynthesis remain poorly known.

Photosynthesis is a prerequisite for cotton yield (Tissue et al., 2010; Wu et al., 2019). Previous research has mainly focused on the effects of photosynthetic physiology in cotton plants (Hu et al., 2016; Shah et al., 2021), winter wheat (Jiang et al., 2004), maize (Wang et al., 2021) and other crops. However, there are few studies on the effects of the combined application of OF and CF on cotton leaf photosynthetic physiology and yield under mulch drip irrigation. This study hypothesized that a new strategy of OF application combined with reduced CF could improve photosynthetic performance and thus increase cotton yield. The main objectives of this study were to 1) investigate dynamic changes in leaf photosynthetic traits and the spatial and temporal distribution of soil organic matter content; 2) determine the relationship between cotton yield, photosynthetic performance, and soil organic matter content; and 3) determine how application of organic liquid fertilizer with reduced application of chemical fertilizer improves cotton yield. The tested hypothesis was that OF combined with reduced CF would improve cotton yield through leaf physiological functioning. Our study is significant for validating the performance of integrated nutrient management through organic and chemical fertilization in cotton, thereby helping farmers optimize cotton production while significantly minimizing conventional fertilizer requirements.

## 2 Materials and methods

### 2.1 Experimental area and soil characteristics

**Figure 1:**
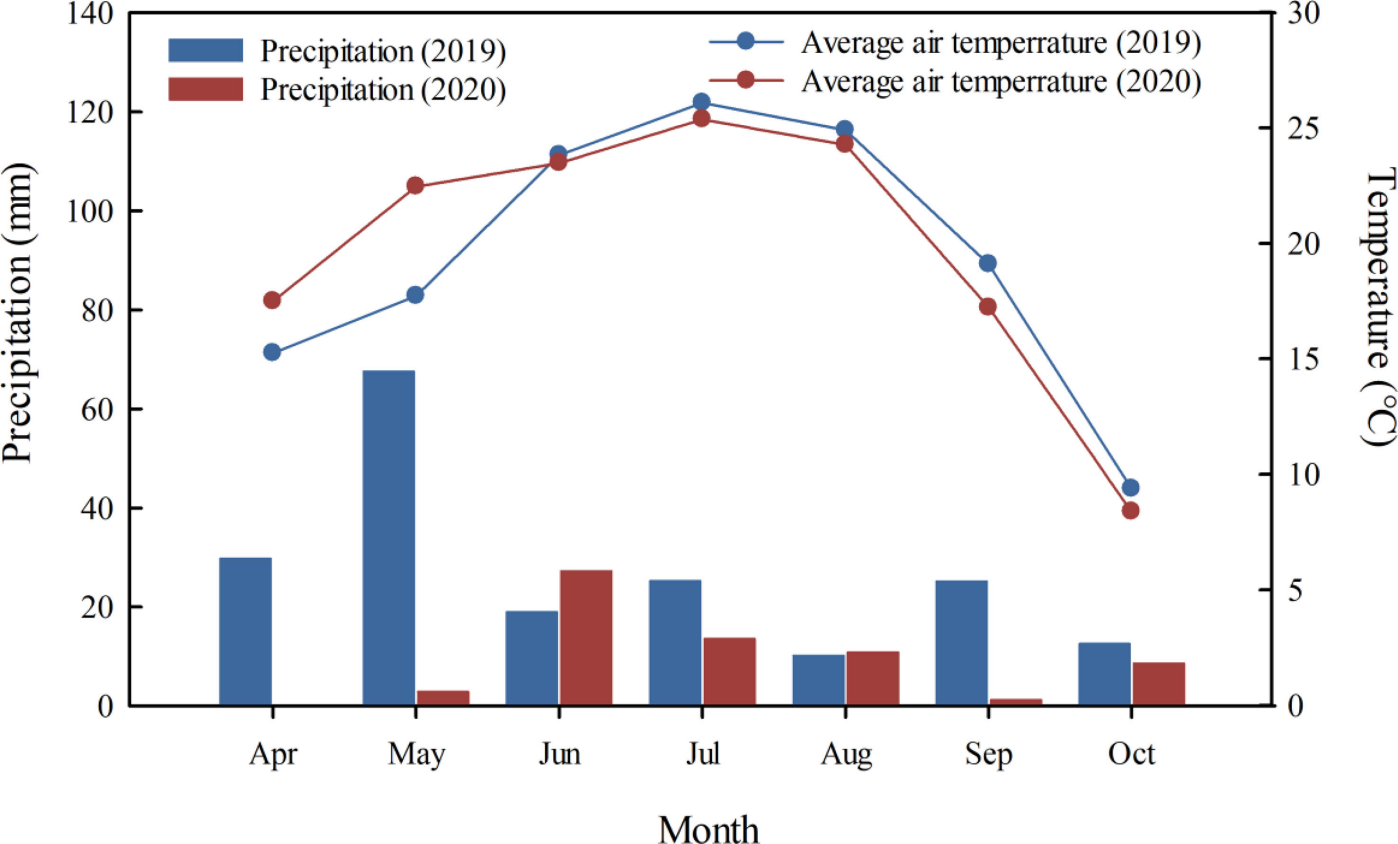
Monthly weather summaries characterizing the 2019 and 2020 cotton growth seasons at the experimental station in Shihezi, Xinjiang, China.

The experiments were conducted during 2019−2020 at the Shihezi Experimental Station for Crop Water Use of the Ministry of Agriculture (Shihezi, Xinjiang, China; 45°38′N latitude, 86°09′E longitude). The cotton cultivar Xinluzao 74 (*Gossypium hirsutum* L.) was sown for two seasons on April 18, 2019, and April 13, 2020. The 20−cm topsoil layer had a sandy loam texture containing 15.0 g kg^−1^ SOM, 42.2 mg kg^−1^ alkali−hydrolysable N, 19.8 mg kg^−1^ P_2_O_5_, 274.3 mg kg^−1^ K_2_O, pH 7.9 and 567 μS cm^−1^ EC. The meteorological data in the 2019 and 2020 cotton-growing seasons are shown in **Figure 1**.

### 2.2 Experimental design and crop management

The experiment was conducted with a randomized block design with six treatments and three replications. The treatments included 100% chemical fertilizer (CF) as a control and the combined application of organic liquid fertilizer (OF) with CF in various ratios. The OF_0.6_, OF_0.8_, OF_1.0_, OF_1.2_ and OF_1.4_ treatments had adjusted ratios of N, P_2_O_5_, and K_2_O. These six treatments were s follows (**Table 1**):

**Table 1:** Ratios of N, P_2_O_5_ and K_2_O for each treatment.

Treatment	Description	N, P and K from CF			N, P and K from OF			Total OF (kg ha^-1^)
		N (kg ha^-1^)	P_2_O_5_ (kg ha^-1^)	K_2_O (kg ha^-1^)	N (kg ha^-1^)	P_2_O_5_ (kg ha^-1^)	K_2_O (kg ha^-1^)	
CF	100% CF	228	131	95	0	0	0	0
OF_0.6_	OF+60% CF	137	78	57	31	2	75	1329.1
OF_0.8_	OF+80% CF	182	104	76	31	2	75	1329.1
OF_1.0_	OF+100%CF	228	131	95	31	2	75	1329.1
OF_1.2_	OF+120%CF	273	157	114	31	2	75	1329.1
OF_1.4_	OF+140%CF	319	183	133	31	2	75	1329.1

**Figure 2:**
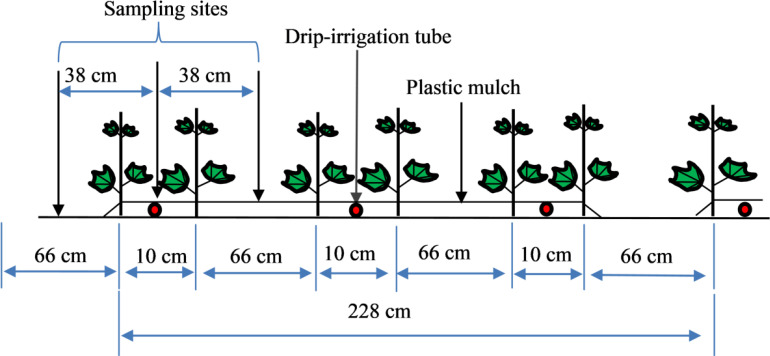
Schematic diagram of the planting mode and sampling site.

Each experimental plot was 68.4 m^2^. The CFs used in this study included urea (46.0% N), monoammonium phosphate (12.0% N and 61.0% P_2_O_5_) and potassium sulfate (50.0% K_2_O). In this study, the OF was organic wastewater with a water-soluble organic matter content of 20.8% and an extremely low heavy metal content. The main properties of the OF are listed in **Table 2**. Cotton was grown under a mulch drip irrigation system. The mulch was 2.28 m wide, with six rows in each mulch sheet (row spacings of 10 cm, 66 cm, 10 cm, 66 cm, 10 cm, and 66 cm, respectively; **Figure 2**). The planting density was 20.1×10^4^ plants ha^−1^. The quantity of irrigation water applied was 4350 m^3^ ha^−1^ (Yao et al., 2016), and all treatments were applied as topdressing under drip irrigation at seeding, squaring, flowering and boll setting (**Table 3**). Other field management parameters were conducted according to local agricultural practices.

**Table 2:** Properties of the organic liquid fertilizer used in this study.

Organic matter (g L^−1^)	Humic acid (g L^−1^)	Macro elements (g L^−1^)			Medium trace elements (g L^−1^)							pH	Liquid density (g mL^−1^)	Microbial flora *Bacillus subtilis* (g L^−1^)	Heavy metals (%)		
		N	P	K	Mo	B	Cu	Mn	Fe	Zn	Ca				Pb	Cd	Cr
208.1	22.1	23.4	1.7	56.6	0.08	0.2	6×10^−8^	0.02	0.1	0.4	3.3	7.2	1.21	2×10^-8^	2.5×10^−4^	1.8×10^−5^	4.7×10^−5^

**Table 3:** Irrigation and fertilizer schedule for the two cotton seasons from 2019 to 2020.

Growth stage	Treatment	N (kg ha^−1^)	P_2_O_5_ (kg ha^−1^)	K_2_O (kg ha^−1^)	Organic liquid fertilizer (L ha^−1^)	Irrigation (m^3^ ha^−1^)
Seeding	CF	9	4.4	1.5	0	300
	OF_0.6_	5.4	2.7	0.9	46.9	300
	OF_0.8_	7.2	3.6	1.2	46.9	300
	OF_1.0_	9	4.4	1.5	46.9	300
	OF_1.2_	10.8	5.4	1.8	46.9	300
	OF_1.4_	12.6	6.3	2.1	46.9	300
Squaring	CF	74.3	40.5	20.3	0	1200
	OF_0.6_	44.6	24.3	12.2	423.3	1200
	OF_0.8_	59.4	32.4	16.2	423.3	1200
	OF_1.0_	74.3	40.5	20.3	423.3	1200
	OF_1.2_	89.1	48.6	24.3	423.3	1200
	OF_1.4_	104	56.7	28.4	423.3	1200
Flowering and boll setting	CF	144.4	85.5	73.5	0	2850
	OF_0.6_	86.6	51.3	44.1	858.9	2850
	OF_0.8_	115.5	68.4	58.8	858.9	2850
	OF_1.0_	144.4	85.5	73.5	858.9	2850
	OF_1.2_	173.3	102.6	88.2	858.9	2850
	OF_1.4_	202.1	119.7	102.9	858.9	2850
Total	CF	227.7	130.4	95.3	0	4350
	OF_0.6_	136.6	78.3	57.2	1329.1	4350
	OF_0.8_	182.1	104.4	76.2	1329.1	4350
	OF_1.0_	227.7	130.4	95.3	1329.1	4350
	OF_1.2_	273.2	156.6	114.3	1329.1	4350
	OF_1.4_	318.7	182.7	133.4	1329.1	4350

### 2.3 Observations

#### 2.3.1 Soil organic matter

At cotton harvesting, soil samples (0~20, 20~40, 40~60, 60~80, 80~100 cm) were collected in each plot. These samples were taken in the vertical direction below the drip irrigation tube and 38 cm from the left and right of the drip irrigation tube in the horizontal direction (**Figure 2**). These samples were air dried, passed through a 0.25-mm mesh sieve and stored at room temperature. Based on the principle of classical potassium dichromate oxidation−outer heating, SOM was determined by oxidation−reduction titration with furnace digestion (Sims et al., 1971).

#### 2.3.2 Chlorophyll content

Cotton leaves (functional leaves) at the initial flowering stage (IF), full flowering stage (FF), full boll stage (FB), late full boll stage (LFB) and boll opening stage (BO) were sampled. Small discs (8.5 mm in diameter) were removed from harvested marked leaves using a hole punch and extracted with 80% acetone solution. Cotton leaf Chl content was determined using a UV−2041 spectrophotometer at wavelengths of 663, 645 and 470 nm with 80% acetone as a blank control. The Chl content was calculated according to the method of Lichtenthaler and Wellburn (1983).

#### 2.3.3 Leaf area

To assess LA, samples were collected at the IF, FF, FB, LFB and BO. The whole-plant LA was determined using the punching method. In each treatment, 3~4 representative plants were sampled, and the leaves of individual plants were punched into 40 pieces with a 15-mm punch. These samples were placed in an envelope, dried at 105°C for 30 min and then dried at 80°C to a constant weight. LA was calculated as follows (Feng and Shi, 2005):


(1) $$LA=Plant\;density\times Whole\;plant\;leaf\;diameter\times \frac{Area\;of\;40\;pieces}{Dry\;mass\;of\;40\;pieces}$$

#### 2.3.4 Leaf gas exchange parameters

Leaf gas exchange parameters, including net photosynthetic rate (*P*_n_), stomatal conductance (*G*_s_) and transpiration rate (E), were measured from fully expanded leaves (fourth node below the terminal) using a Li−6800 portable photosynthetic apparatus (Li−COR, Lincoln, NE, USA) at the IF, FF, FB, LFB and BO. These measurements were taken on a clear sunny day using a photosynthetic photon flux density of 1800 μmol m^−2^ s^−1^.

#### 2.3.5 Chlorophyll fluorescence parameters

The chlorophyll fluorescence parameters were measured by a MINI−PAM fluorometer at five different growth stages, i.e., IF, FF, FB, LFB and BO, from fully expanded leaves (fourth node below the terminal). After dark adaptation, the initial fluorescence (*F*_0_) and maximum fluorescence (*F*_m_) were measured, and the maximum photochemical efficiency (*F*_v_/*F*_m_) was calculated. Then, the photochemical light was turned on, and the light intensity was stabilized at 1200~1400 μmol m^−2^ s^−1^. The saturating pulse was turned on when the fluorescence signal reached the steady state. The actual fluorescence yield (*F*_t_) and the maximum fluorescence yield (*F*_m’_) under light adaptation were measured, and the light energy capture efficiency (Y(II)) and other parameters were calculated as follows (Schreiber et al, 1995):


(2) $$Photochemical\;efficiency\;of\;PSII:\frac{F_{v}}{F_{m}}=\frac{\left(F_{m}-F_{0}\right)}{F_{m}}$$

(3) $$Efficiency\;of\;PSII\;light\;energy\;capture:Y(II)=\frac{\left(F_{m}^{'}-F_{t}\right)}{F_{m}^{'}}$$

(4) $$Electron\;transport\;rate\;of\;PSII:ETR=\frac{F_{m}^{'}-F_{t}}{F_{m}^{'}}\times PAR\times 0.5\times 0.84$$

(0.5 is the proportion of light energy distribution in PSII and PSI, and 0.84 is the light absorption coefficient of the leaf, PAR = photosynthetically active radiation).


(5) $$Photochemical\;quenching:qP=1-\frac{F_{t}-F_{0}}{F_{m}^{'}-F_{0}}$$

(6) $$Non\;photochemical\;quenching:NPQ=1-\frac{F_{m}-F_{m}^{'}}{F_{m}^{'}}$$

#### 2.3.6 Biological yield, seed yield and economic coefficient

During the cotton harvest (Sep. 22, 2019, and Sep. 26, 2020), 3 representative sampling points (2 m×2.28 m) were selected in each treatment, and the seed cotton yield was calculated using the number of bolls per unit area and single boll weight. Four plants were sampled during BO, and these plants were divided into different organs, i.e., stems, leaves and buds. These samples were put in an envelope, placed in an oven at 105°C for 30 min, and dried at 85°C to obtain a constant weight, and the dry weight was recorded. The biological yield was analyzed and calculated. The economic coefficient was calculated as follows (Gao et al, 1990):


(7) $$Economic\;coefficient=\frac{Seed\;yield}{Biological\;yield}$$

### 2.4 Data analysis

Microsoft Excel 2016 was used for data collation. SPSS 19.0 (IBM Inc., Chicago, IL, USA) software was applied for analysis of variance (ANOVA) and least significant difference (LSD) test at a significance level of 0.05 with a general linear model. Pearson’s correlation analysis and principal component analysis were carried out using the “ggplot2” in R 4.0.4 software (R Core Team 2021). Figures were plotted using Sigmaplot 14.0 (Systat Software Inc., San Jose, CA, USA), Origin 2021and Surfer16.0 (Golden Software Inc., USA). The data are presented as the mean and standard error (SE).

## 3 Results

### 3.1 Soil organic matter

**Figure 3:**
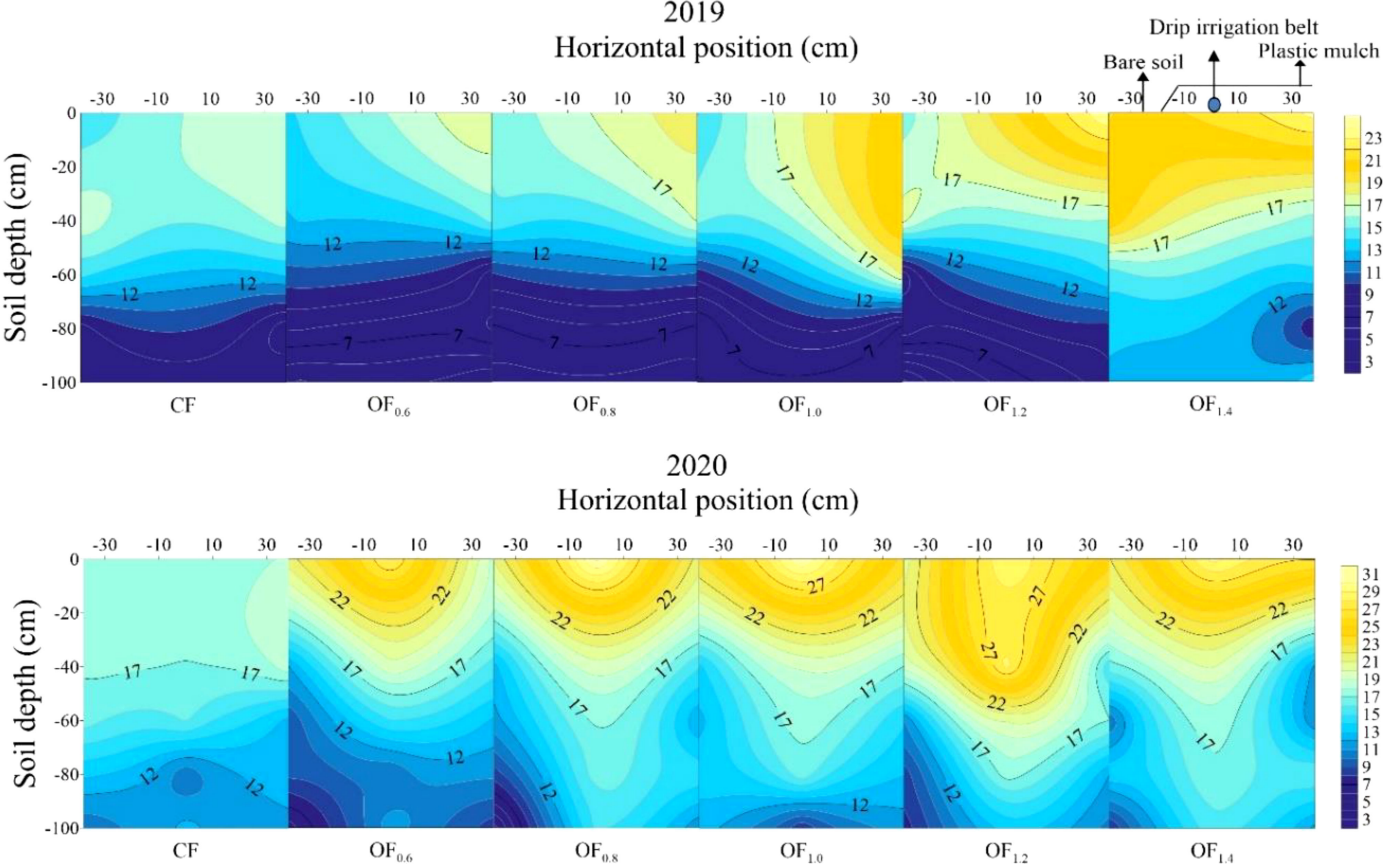
Spatial distribution of SOM (g kg^-1^) in response to organic and chemical fertilizer in 2019−2020.

Compared with the CF treatment, OF evenly increased SOM by 13.4% (**Figure 2**). OF_0.8_, OF_1.0_ and OF_1.2_ resulted in higher SOM contents of 22.7, 22.6 and 25.8 g kg^−1^ at the 0~60 cm soil depth, respectively (**Figure 3**).

### 3.2 Chlorophyll content

**Figure 4:**
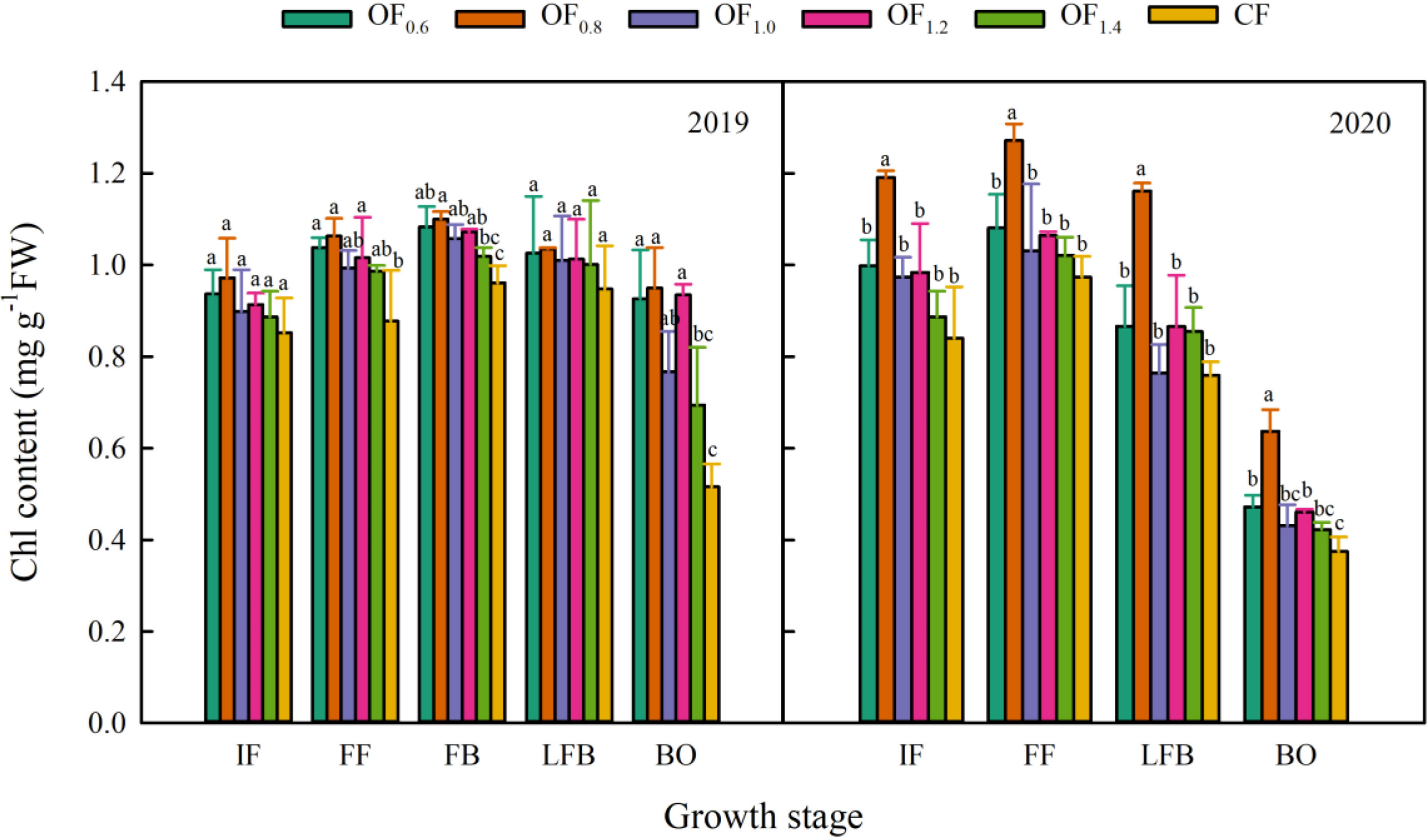
Effect of different fertilization treatments on Chl content (mg g^−1^ FW). Different letters indicate significant differences among treatments at a specific growth stage (P ≤ 0.05).

The Chl content was higher from FF to FB (**Figure 4**), with the following trend: OF_0.8_ > OF_0.6_, OF_1.2_, OF_1.0_ > OF_1.4_, CF. In 2019, there was no significant difference between the OF_0.6_, OF_0.8_, OF_1.0_ and OF_1.2_ treatments, which evenly increased the Chl content by 17.5%, 19.3%, 13.0% and 16.4% compared with CF, respectively. In 2020, the Chl content in the OF_0.8_ treatment was significantly higher than that in the other treatments, and there was no significant difference between the other fertilization treatments and CF (except OF_1.4_ at BO).

### 3.3 Leaf area

**Figure 5:**
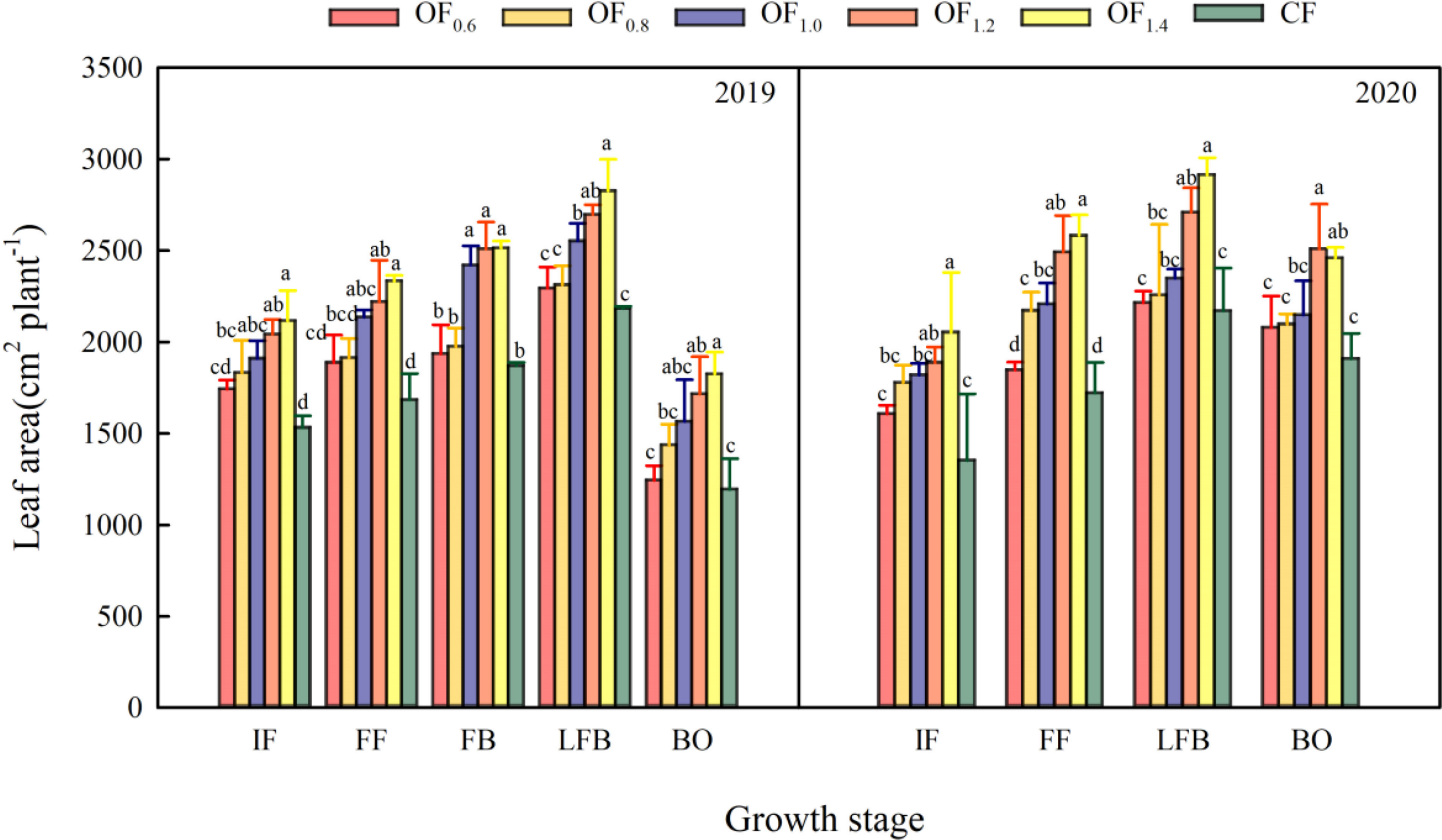
Effect of different fertilization treatments on leaf area (cm^−2^ plant^−1^). Different letters indicate significant differences among treatments at a specific growth stage (P ≤ 0.05).

The cotton plant LA first increased and then decreased with plant growth (**Figure 5**). The LA under OF combined with CF was 19.2% higher than that under the CF treatment, but no significant difference between the OF_0.6_ and OF_0.8_ treatments was noted in either year. In 2019, LA increased by 20.2%, 24.6% and 27.6% for the OF_1.0_, OF_1.2_ and OF_1.4_ treatments compared with CF, respectively. In 2020, the OF_1.2_ and OF_1.4_ treatments had 25.8% and 28.9% higher LAs than the CF treatment.

### 3.4 Leaf gas exchange parameters

**Figure 6:**
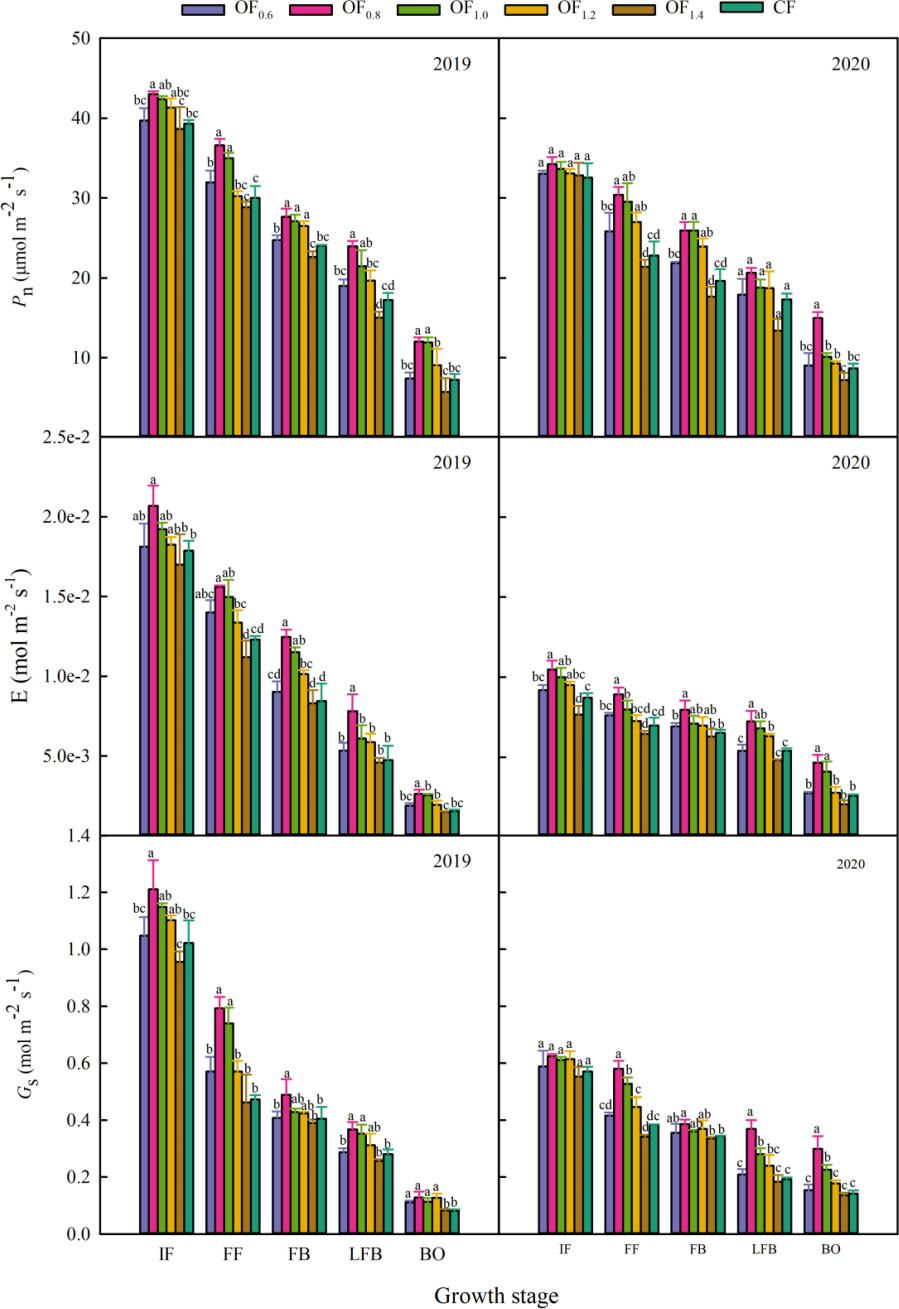
Effect of different fertilization treatments on the gas exchange parameters of cotton. Different letters indicate significant differences among treatments at a specific growth stage (P ≤ 0.05).

Cotton leaf photosynthetic performance parameters, i.e., *P*_n_, *G*_s_ and E, increased first and then decreased with the increase in the ratio of CF combined with OF (**Figure 6**). Compared with the CF treatment, application of OF_0.8_ and OF_1.0_ resulted in 22.0% and 16.4%, 27.4% and 20.4%, and 26.8% and 20.9% higher *P*_n_, E, and *G*_s_ values, respectively. Compared with CF, under the OF_1.2_ treatment, *P*_n_, E, and *G*_s_ increased by 9.6%, 10.6%, and 14.3%, respectively. The leaf gas exchange parameters of the OF_0.6_ and OF_1.4_ treatments were not significantly different from those of the CF treatment.

### 3.5 Chlorophyll fluorescence parameters

**Figure 7:**
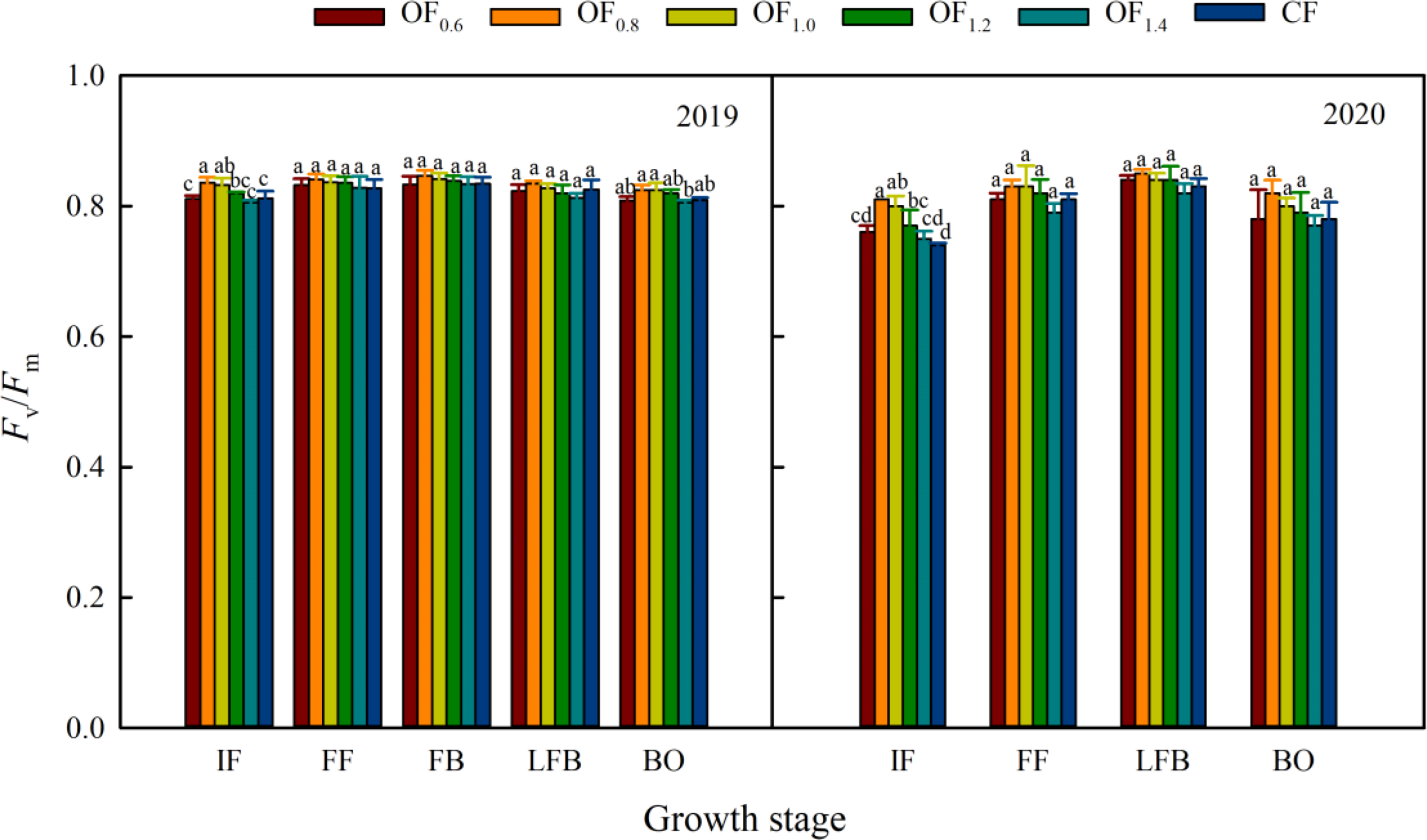
Effect of different fertilization treatments on *F*_v_/*F*_m_. Different letters indicate significant differences among treatments at a specific growth stage (P ≤ 0.05)

**Figure 8:**
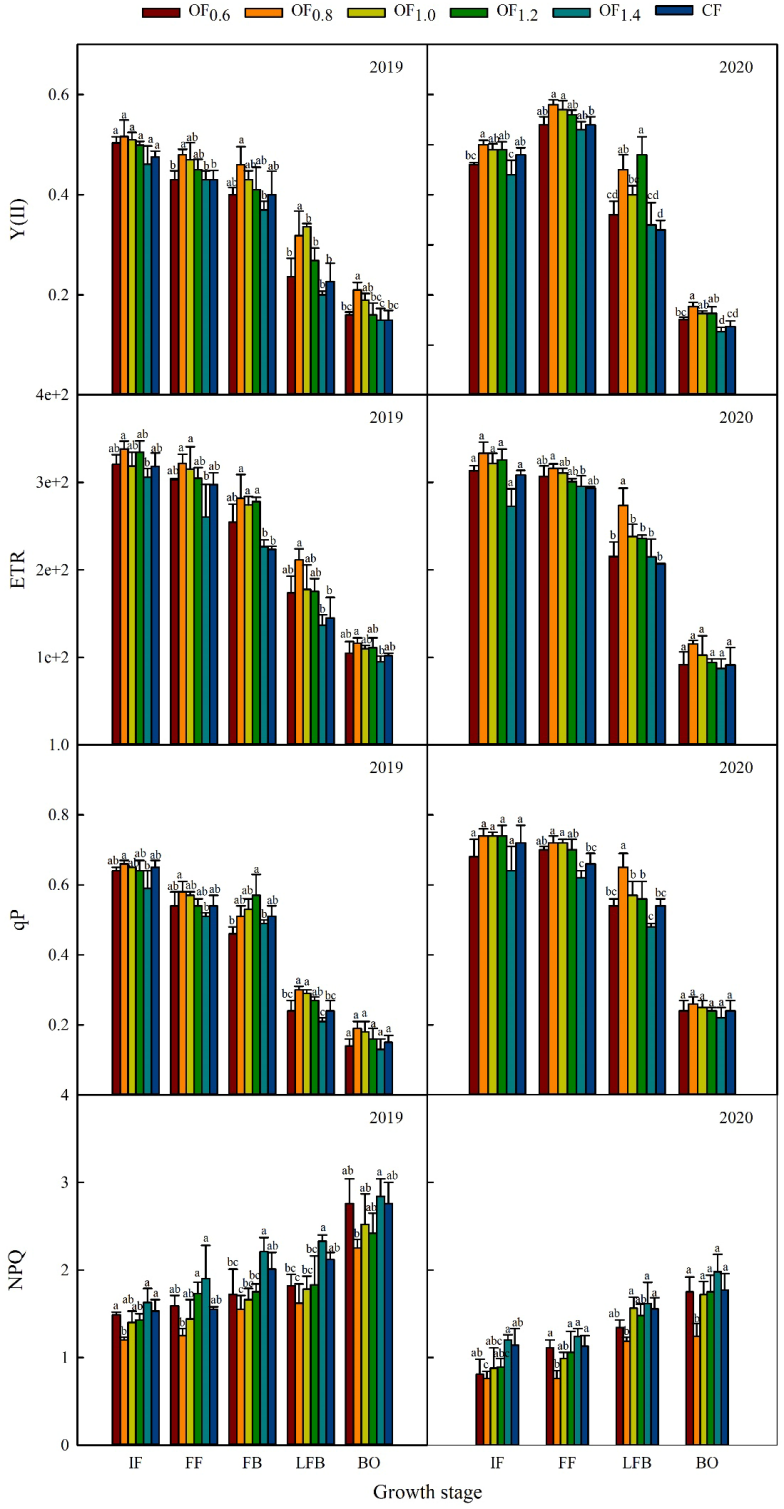
Effect of different fertilization treatments on chlorophyll fluorescence parameters. Different letters indicate significant differences among treatments at a specific growth stage (P ≤ 0.05).

Cotton leaf Chl fluorescence attributes were significantly affected by OF and CF in both years (**Figures 7**, **8**). *F*_v_/*F*_m_ followed the trend OF_0.8_> OF_1.0_, OF_1.2_> OF_0.6_, and OF_1.4_> CF (**Figure 7**), but there was no significant difference among the treatments from LFB to BO. Compared with the CF treatment, under the OF_0.8_ and OF_1.0_ treatments, *F*_v_/*F*_m_ increased by 6.1% and 4.7%, respectively.

Different letters indicate significant differences among treatments at a specific growth stage (P ≤ 0.05)

The cotton leaf Y(II), ETR and qP first increased and then decreased with the increase in the proportion of CF combined with OF in the following order: OF_0.8_> OF_1.2_, OF_1.0_> CF, OF_0.6_, OF_1.4_ (**Figure 8**). The OF_0.8_ and OF_1.0_ treatments had 16.3% and 12.8% higher Y(II) values (except at LFB in 2019) than the CF treatment. Compared with CF, Y(II) under the OF_1.2_ treatment increased by 10.0%, but there was a significant difference from LFB to BO in 2020. Under the OF_0.8_ treatment, ETR and qP increased by 15.3% and 9.8% compared with CF, respectively. Significant differences from LFB to BO in 2019 and at LFB in 2020 were noted (**Figure 7**). The cotton leaf nonphotochemical quenching (NPQ) trend among the treatments was as follows: OF_1.4_, CF > OF_0.6_, OF_1.0_, OF_1.2_ > OF_0.8_. NPQ increased by 25.5% for the OF_0.8_ treatment compared with CF in 2019.

### 3.6 Biological yield, seed yield and economic coefficient

**Figure 9:**
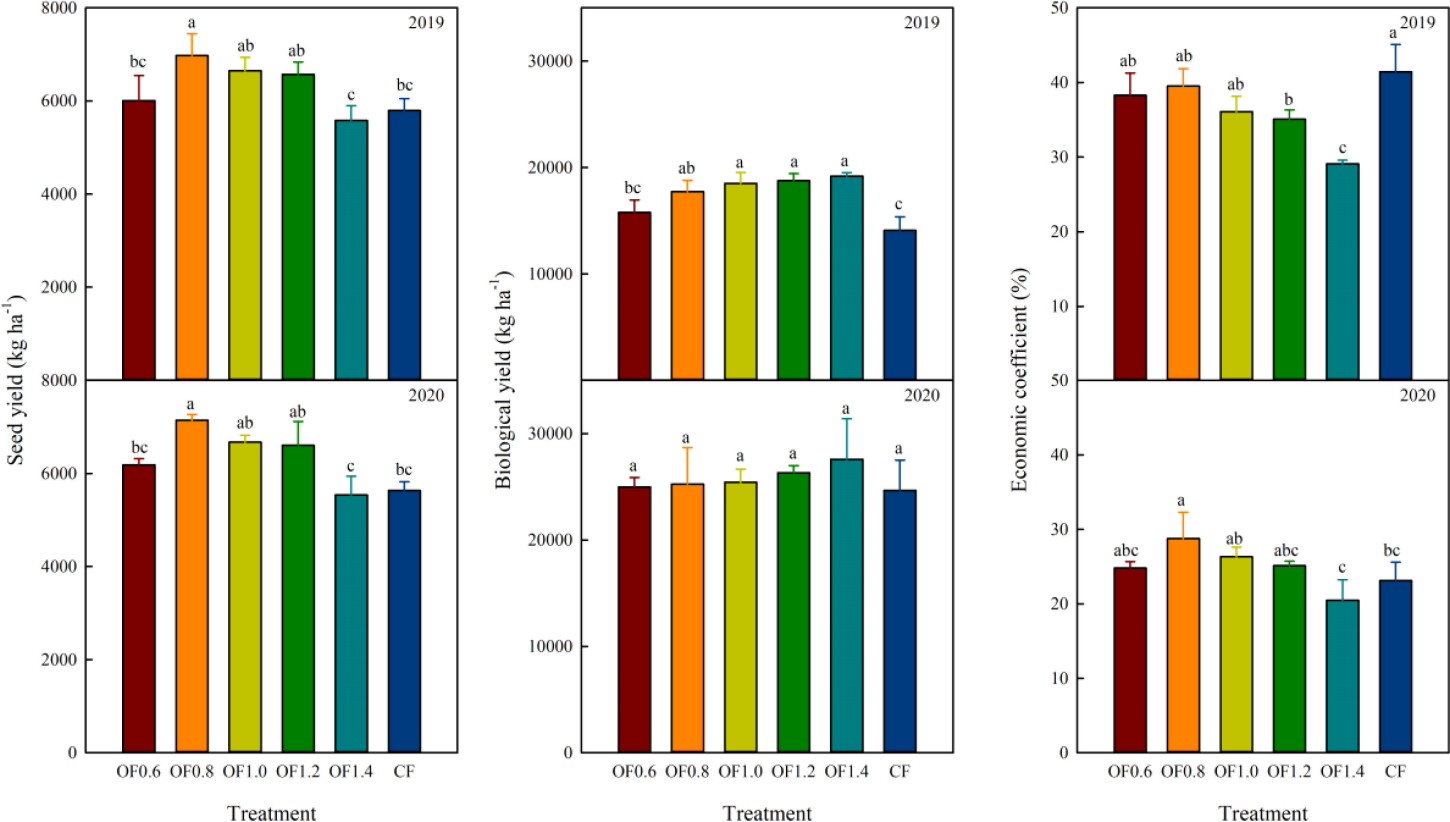
Effect of different fertilization treatments on the biological yield (kg ha^−1^), seed yield (kg ha^−1^) and economic coefficient (%) of cotton. Different letters indicate significant differences among treatments at a specific growth stage (P ≤ 0.05).

Both fertilizer types significantly affected the biological yield, seed yield and economic coefficient of cotton plants in both years (**Figure 9**). Compared with CF, the OF_0.8_, OF_1.0_ and OF_1.2_ treatments increased seed yield by 17.0%, 14.3% and 13.3%, respectively. No significant differences among the CF, OF_0.6_ and OF_1.4_ treatments were noted. The cotton biological yield for the OF_0.8_, OF_1.0_, OF_1.2_ and OF_1.4_ treatments increased by 20.5%, 23.8%, 24.9% and 26.6% compared with CF, respectively. No significant difference was found between the CF and OF_0.6_ treatments. Significant differences among the treatments were observed in 2019, where the economic coefficient of OF_0.8_ was increased by 19.6% compared with that of CF. No significant differences among the treatments in 2020 were noted. Compared with CF, the economic coefficient of the OF_1.4_ treatment decreased by 20.7% in 2019–2020.

### 3.7 Pearson’s correlation analysis

**Figure 10:**
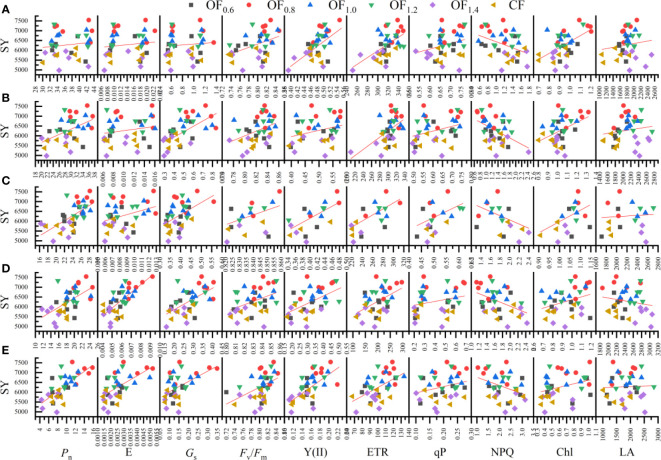
Linear regression relationship between seed yield (SY) and measured parameters (i.e., Pn, Gs, E, Y(II), ETR, qP, NPQ, Fv/Fm, Chl and LA) at initial flowering **(A)**, full flowering **(B)**, full boll **(C)**, late full boll **(D)**, and boll opening **(E)** stages.

**Figure 11:**
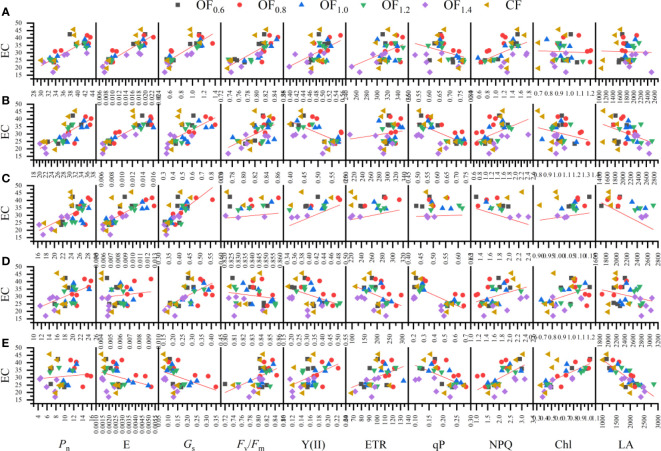
Linear regression relationship between economic coefficient (EC) and measured parameters (i.e., Pn, Gs, E, Y(II), ETR, qP, NPQ, Fv/Fm, Chl and LA) at initial flowering **(A)**, full flowering **(B)**, full boll **(C)**, late full boll **(D)**, and boll opening **(E)** stages.

**Figure 12:**
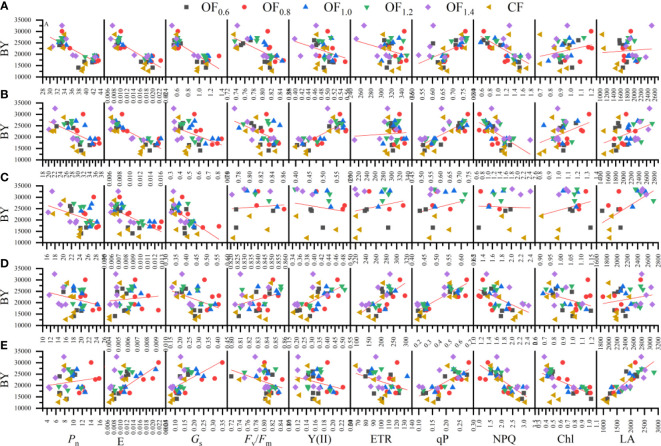
Linear regression relationship between biological yield (BY) and measured parameters (i.e., Pn, Gs, E, Y(II), ETR, qP, NPQ, Fv/Fm, Chl and LA) at initial flowering **(A)**, full flowering **(B)**, full boll **(C)**, late full boll **(D)**, and boll opening **(E)** stages.

The Pearson correlation coefficients of different parameters is shown in **Figures 10**–**12**. Seed yield (**Figure 10**) was positively correlated with Chl content from IF to FF; with *P*_n_ and *F*_v_/*F*_m_ from FF to BO; with ETR and Y(II) at IF, FB and BO; with *G*_s_ at FF; and with *G*_s_ and E from LFB to BO. Seed yield was significantly negatively correlated with qP at FB. Biological yield was significantly positively correlated with qP from IF to FF and from LFB to BO; with Y(II) at FF and LFB; with ETR at LFB; and with LA at BO.

Biological yield (**Figure 12**) was significantly negatively correlated with *P*_n_ from IF to FF; with E and *G*_s_ from IF to FB; with *F*_v_/*F*_m_ at IF, FF and BO; with NPQ from IF to FF and from LFB to BO; and with Chl content at BO. The economic coefficient (**Figure 11**) was positively correlated with *P*_n_ and E from IF to FB; with *G*_s_ from IF to LFB; with *F*_v_/*F*_m_ at IF, FF and BO; with NPQ at FF and BO; with Chl from LFB to BO; and with ETR at BO. The economic coefficient was negatively correlated with qP at FF, LFB and BO; with Y(II) at FF; and with LA at FB and BO.

### 3.8 Principal component analysis

**Figure 13:**
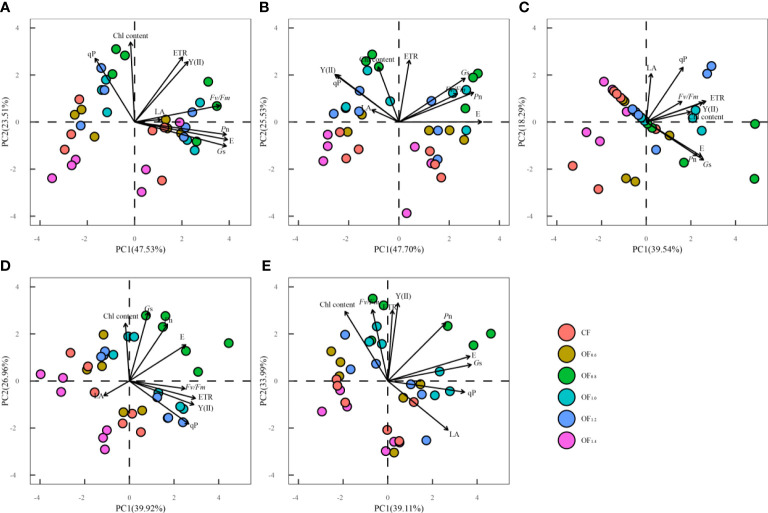
Principal component analysis among measured parameters (i.e., *P*_n_, *G*_s_, E, Y(II), ETR, qP, NPQ, *F*_v_/*F*_m_, Chl and LA) at initial flowering **(A)**, full flowering **(B)**, full boll **(C)**, late full boll **(D)**, and boll opening **(E)** stages.

Principal component analysis of LA, gas exchange parameters, fluorescence parameters and other measurement parameters of cotton was performed at different growth stages (**Figure 13**). At the initial flowering stage, the contribution rates of the first (PC1), second (PC2), and third (PC3) principal components were 47.5, 23.5, and 12.1%, respectively. The cumulative contribution of the first three principal components was 83.2%. The largest loading values on PC1 were those of E, *G*_s_, and *P*_n_ at 0.95, 0.94, and 0.94, respectively, followed by those of *F*_v_/*F*_m_, Y(II), and ETR, and there was a negative correlation with Chl content and qP, indicating that cotton has strong photosynthetic capacity at the initial flowering stage. The maximum loading value on PC2 was that of Chl content at 0.82, followed by that of ETR, qP, and Y(II). This indicates that photosynthesis dominates at the initial flowering stage. Moreover, the OF_0.8_, OF_1.0_, and OF_1.2_ treatments contributed more to photosynthesis.

The contribution rates of the first, second, third, and fourth (PC4) principal components during the full flowering stage were 43.7%, 25.5%, 11.4%, and 6.9%, respectively, with a cumulative contribution rate of 87.6% for the four principal components. The largest loading values on PC1 were those of E, *G*_s_, and *P*_n_ at 0.96, 0.86, and 0.77, respectively, followed by those of *F*_v_/*F*_m_ and ETR, and there was a negative correlation with LA, Y(II), Chl content and qP. The maximum loading value on PC2 was that of ETR at 0.78, followed by that of Chl content. In PC2, different fertilization treatments can be clearly distinguished, indicating that reducing fertilizer application does not result in nutrient stress on cotton.

The contribution rates of the first, second, third, and fourth principal components during the full boll stage were 39.5%, 18.3%, 13.5%, and 11.2%, respectively, with a cumulative contribution rate of 82.5% for the four principal components. The maximum loading value on PC1 was that of ETR at 0.79, followed by those of E and *G*_s_ at 0.73 and 0.76, respectively. The maximum loading value on PC2 was that of qP at 0.68, followed by that of LA, and there was a negative correlation with *P*_n_, E, and *G*_s_. The contribution rates of the first, second, third, and fourth principal components during the later full boll stage were 39.9%, 26.9%, 11.9%, and 7.1%, respectively, with a cumulative contribution rate of 85.9% for the four principal components. PC1 can clearly distinguish among different treatments, and the maximum loading value on PC1 was that of ETR at 0.87, followed by that of Y(II), qP, and E. The loading values of *G*_s_, Chl content, and *P*_n_ on PC2 were relatively high, and there was a negative correlation with *F*_v_/*F*_m_, LA, Y(II), ETR, and qP. This indicates that ETR is the main parameter reflecting photosynthetic capacity from the full boll stage to the later full boll stage.

The contribution rates of the first, second, third, and fourth principal components of each parameter during the boll opening stage were 39.1%, 33.9%, 7.1%, and 5.8%, respectively, with a cumulative contribution rate of 86.0% for the four principal components. The maximum loading value on PC1 was that of *G*_s_ at 0.92, followed by that of E, qP and LA. The maximum loading value on PC2 was that of Y(II) at 0.82, followed by that of *F*_v_/*F*_m_, ETR and Chl content.

## 4 Discussion

Long−term and excessive use of CF may not sustain high crop yields and lead to environmental pollution, such as greenhouse gas emissions to the atmosphere (Khan et al., 2017a). In this study, compared with the CF treatment, higher biological yields, seed yield (OF_0.8_) and economic coefficient (OF_0.8_) might have been achieved because of the combined effect of OF and CF application, enhancing soil nutrient availability and improving nutrient absorption and utilization by cotton plants (Saikia et al., 2015). This result may also have occurred because the reduced CF amount promoted leaf functioning, i.e., photosynthetic characteristics, which further increased aboveground biomass formation and its transition to reproductive organs, resulting in a higher cotton yield (Khan et al., 2017b; Ahangera et al., 2021; Shi et al., 2021). This suggests that under mulch drip irrigation systems, the application of OF can ensure a high cotton seed yield and economic coefficient due to enhanced cotton biomass accumulation.

Moreover, SOM is essential for maintaining ecosystem function and agricultural sustainability (Raiesi, 2021). In this study, SOM significantly increased under OF application compared with the CF treatment. This finding suggests that OF can improve soil quality and increase crop yield (Urra et al., 2020). In this study, OF (OF_0.8_, OF_1.0_ and OF_1.2_) resulted in a higher SOM content at the 0−60 cm soil depth. Previous studies showed that the SOM content reached 22.84 g kg^−1^ after the long-term application of traditional organic fertilizer from 1992 to 2016 (Chen et al., 2022). However, OF was supplied in both years, which resulted in SOM values of 22.7−25.8 g kg^−1^ possibly due to the following reasons: 1) Under OF, the water−soluble organic matter content was up to 20.8%. The decomposition rate of organic matter in the organic waste liquid was fast, which was beneficial for rapidly increasing SOM (Mooy et al., 2019); 2) Small amounts of organic waste liquid and CF were applied several times throughout the whole cotton growth period to avoid soil loss and volatilization of nutrients. This application scheme further favored the accumulation of organic matter in the soil as well as an enhancement in plant nutrients (Agbede et al., 2010). Hence, under mulch drip irrigation systems, the application of OF can induce a suitable soil nutrient environment for the efficient production of cotton.

Furthermore, crop yield formation is also regulated by photosynthetic performance, Chl content and photosynthetic area (Santos et al., 2013; Wang et al., 2015; Wang et al., 2016; Shah et al., 2021). In this study, compared with the CF treatment, the OF_0.8_ treatment resulted in a higher Chl content, *P*_n_, *G*_s_ and E. The increases in these parameters resulted from the application of OF, which released more soil carbon dioxide, which regulates stomatal opening and consequently leads to a high photosynthetic rate (Dębska et al., 2016). Second, the use of OF can increase the nitrogen content of plant functional leaves, which in turn improves the activities of photosynthetic carbon assimilation enzymes (Rubisco), leading to a higher photosynthetic rate and cotton yield (Grassi et al., 2010; Sepehri and Sanavy, 2003). In this study, LA increased with increasing ratios of OF, possibly because the combination of OF with excessive CF resulted in a larger LA. This further results in severe canopy shading, greatly reducing the ability of the canopy to intercept light energy and thus inhibiting the net photosynthetic performance of cotton plants (Hueso et al., 2021). The photosynthetic performance of the cotton leaves did not increase as the ratio of OF combined with CF increased, suggesting that an appropriate proportion of OF combined with CF can create a good light environment by maintaining a reasonable LA, high Chl content and high photosynthetic performance.

Additionally, Chl fluorescence plays a unique role in measuring photosynthesis and in the absorption, transmission, dissipation and distribution of light energy by the photosystem (Sinsawat et al., 2004; Dorta-Santos et al., 2020). The maximum photochemical efficiency (*F*_v_/*F*_m_) is an important indicator of whether plants are under stress; a value above 0.8 indicates that the plants are not under stress, and a slight decline in the value of this parameter may correspond to a light protection mechanism (Narayan et al., 2020). At IF in 2020, the *F*_v_/*F*_m_ values of the CF, OF_0.6_, OF_1.2_, and OF_1.4_ treatments began to decline to less than 0.8, indicating that cotton leaves in these four treatments experienced photoinhibition, and the activity of the PSII reaction center was significantly reduced.

However, the *F*_v_/*F*_m_ values of OF_0.8_ and OF_1.0_ remained at approximately 0.8, which indicates that these OF treatments better promote the PSII primary light energy conversion efficiency of cotton plant leaves; that is, the captured light energy is converted into chemical energy, which is conducive to improving the photosynthetic capacity of cotton (Zhang et al., 2013). This suggests that the combination of OF and CF (OF_0.8_, OF_1.0_ and OF_1.2_ treatments) could improve the drought resistance of cotton. Compared with the CF treatment, the OF treatments improved the Y(II), ETR and qP of the cotton. In addition, the OF_0.8_ and OF_1.0_ treatments significantly increased Y(II), ETR and qP in this study. This indicates that the application of CF alone and the OF_1.4_ treatment may decrease the PSII quantum yield, blocking electron transfer in the PSII reaction and decreasing the photochemical activity of the PSII reaction center. An appropriate application of OF and CF can delay cotton plant senescence, which results in increased light energy utilization efficiency in crop leaves (Ikeuchi et al., 2014).

Improving the photosynthetic capacity (photosynthetic rate) can increase seed cotton yield under limited drip irrigation systems (Gao et al., 2021). In addition, a higher leaf *F*_v_/*F*_m_ can promote the photosynthetic capacity of reproductive organs and ultimately build high-yield cotton with optimum fruiting nodes (Zhen et al., 2020). It has been shown that improving the leaf photosynthetic capacity in the later stage can guarantee a high seed cotton yield (Li et al., 2006), as evidenced in this study as well. Photosynthetic performance and soil nutrients are the key indicators for evaluating irrigation and fertilization management benefits (Luo et al., 2023). The result of PCA principal component analysis showed that multiple correlations between yield and PSII parameters, photosynthetic performance parameters are the main factor responsible for differences among treatments (Rodríguez et al., 2023). In addition, previous studies have also demonstrated that photosynthetic parameters such as *F*_v_/*F*_m_, *F*_v_/*F*_o_, ETR, Chl a, and Chl b have a significant positive impact on crop yield (Li et al., 2023). In this study, photosynthetic potential was main factor causing differences in the early growth stages of cotton. OF_0.8_, OF_1.0_, and OF_1.2_ treatments enhanced photosynthetic performance. In the late growth stage (from LFB to BO stage), ETR was the key driver reflecting photosynthetic capacity. OF combined with an appropriate amount of CF can improve crop yield due to the optimized relationship between photosynthetic capacity (*P*_n_, *G*_s_ and Y(II)) and crop yield formation and can promote the transport of photosynthetic products to the grains, resulting in an increased seed yield (Guo et al., 2020). These findings indicate that OF combined with CF is a promising fertilization method for efficient cotton production under a mulch drip irrigation system.

Overall, our study elucidates that an optimum combination of organic and conventional fertilizer at a reduced rate could be an improved strategy to optimize nutrient uptake, enhance soil fertility and productivity and harness the maximum leaf photosynthetic efficiency. An integrated approach not only promotes better growth and higher yield but also minimizes the potential off-farm loss of chemical inputs, thereby reducing collateral environmental pollution. Further investigation with different organic inputs under various management systems can provide farmers with further strategies to utilize integrated nutrient management practices for cotton production.

## 5 Conclusion

In this study, OF combined with 80% CF increased seed cotton yield by 5.6−21.2% and economic coefficient by 8.4−27.6%. Furthermore, OF combined with 80% CF resulted in a higher Chl content, *P*_n_, E, *G*_s_, Y(II), ETR, and qP but decreased NPQ. The seed cotton yield and economic coefficient were positively correlated with *P*_n_, *F*_v_/*F*_m_, *G*_s_ and Y(II) from FB to BO. Improvements in these attributes further resulted in a higher economic coefficient and seed cotton yield in both years. Hence, OF (dissolved organic matter content > 20.8%, with a total application amount of 1329 kg ha^−1^) combined with 80% CF (N−P_2_O_5_−K_2_O: 182−104−76 kg ha^−1^) is a promising option to improve leaf photosynthetic performance and light energy utilization efficiency, resulting in a higher seed cotton yield and economic coefficient. Our study validated the superior performance of integrated nutrient management in cotton using organic liquid fertilizer in combination with chemical fertilization at a reduced rate, thereby providing farmers with alternative strategies for cotton management to support clean production. Our results provide new insights into sustainable cotton production using integrated crop management strategies under mulch drip irrigation.

## Data availability statement

The original contributions presented in the study are included in the article/Supplementary Material. Further inquiries can be directed to the corresponding authors.

## Author contributions

XS, JW, and HL conceived the main idea and designed the experiment. XH, AK, JN edited and provided suggeston in this article. NL, JL and helped in data collection. FS and YT helped in labortory analysis. All authors contributed to the article and approved the submitted version.

## References

[B1] Agbede T. M. Oladitan T. O. Alagha S. A. Ojomo A. O. Ale M. O. (2010). Comparative evaluation of poultry manure and NPK fertilizer on soil physical and chemical properties, leaf nutrient concentrations, growth and yield of yam (Dioscorea rotundata Poir) in Southwestern Nigeria. World J. Agric. Sci. 6, 540–546. Available at: https://www.mendeley.com/catalogue/e2ef0061-2a30-30cf-b39d-083e332ceed6/.

[B2] Ahangera M. A. Qi M. Huang Z. Xu X. Begum N. Qin C.. (2021). Improving growth and photosynthetic performance of drought stressed tomato by application of nano–organic fertilizer involves up–regulation of nitrogen, antioxidant and osmolyte metabolism. Ecotoxicol. Environ. Saf. 216, 112195. doi: 10.1016/j.ecoenv.2021.112195 33823368

[B3] Arshad A. Raza M. A. Zhang Y. Zhang L. Wang X. Ahmed M.. (2021). Impact of climate warming on cotton growth and yields in China and Pakistan: a regional perspective. Agriculture 11, 97. doi: 10.3390/agriculture11020097

[B4] Chen H. N. Zhou H. P. Wen Y. L. Xiang Y. Cheng M. (2022). Ecological stoichiometric characteristics of soil nutrients and eco-enzymatic activities under different long-term fertilizations in a cinnamon soil. Plant Nutr. Fert. Sci. 28, 972–983. doi: 10.11674/zwyf.2021505

[B5] Dębska B. Długosz J. Piotrowska–Długosz A. Banach–Szott M. (2016). The impact of a bio–fertilizer on the soil organic matter status and carbon sequestration—results from a field–scale study. J. Soil Sediment. 16, 2335–2343. doi: 10.1007/s11368-016-1430-5

[B6] Dorta-Santos M. A. Barriola I. Wassner D. F. Ploschuk E. L. (2020). Photosynthesis, fluorescence and mesophyll conductance responses to increasing salinity levels in Jatropha curcas at early vegetative stages. J. Agron. Crop Sci. 206, 52–63. doi: 10.1111/jac.12373

[B7] Feng D. Shi S. (2005). Research on night measurement method of leaf area. Chin. Agron. Bull. 21, 150–152+155. doi: 10.3969/j.issn.1000-6850.2005.06.043

[B8] Gao Q. Benedict. C. R. Kohel. R. J. (1990). Variation of dry matter distribution and fiber elongation and weight gain in different varieties of upland cotton bolls. Cott Sci. 2, 52–57. Available at: http://en.cnki.com.cn/Article_en/CJFDTOTAL-MHXB199002008.htm.

[B9] Gao H. Y. Li N. N. Li J. H. Aziz K. Ijaz A. Wang Y. Y.. (2021). Improving boll capsule wall, subtending leaves anatomy and photosynthetic capacity can increase seed cotton yield under limited drip irrigation systems. Ind. Crops Products 161, 113214. doi: 10.1016/j.indcrop.2020.113214

[B10] Grassi G. Meir P. Cromer R. Tompkins D. Jarvis P. G (2010). Photosynthetic parameters in seedlings of Eucalyptus grandis as affected by rate of nitrogen supply. Plant Cell Environ 25, 1677–1688. doi: 10.1046/j.1365-3040.2002.00946.x

[B11] Guo X. J. Xie J. H. Li L. L. Wang J. N. Kang C. R. Peng Z. K.. (2020). Appropriate nitrogen fertilizer rate and organic N ratio for satisfactory photosynthesis and yield of maize in dry farming area of Longzhong, Gansu Province. Plant Nutr. Fert. Sci. 26, 806–816. doi: 10.11674/zwyf.19279

[B12] Hu W. Jiang N. Yang J. S. Meng Y. L. Wang Y. H. Chen B. L.. (2016). Potassium (K) supply affects K accumulation and photosynthetic physiology in two cotton (Gossypium hirsutum L.) cultivars with different K sensitivities. Field Crop Res. 196, 51–63. doi: 10.1016/j.fcr.2016.06.005

[B13] Hueso A. Camacho G. Gómez–Del–Campo M. (2021). Spring deficit irrigation promotes significant reduction on vegetative growth, flowering, fruit growth and production in hedgerow olive orchards (cv. Arbequina). Agr. Water Manage. 248, 106695. doi: 10.1016/j.agwat.2020.106695

[B14] Ikeuchi M. Uebayashi N. Sato F. Endo T. (2014). Physiological functions of PsbS–dependent and PsbS–independent NPQ under naturally fluctuating light conditions. Plant Cell Physiol. 55, 1286–1295. doi: 10.1093/pcp/pcu069 24850835

[B15] Jagadamma S. Lal R. Hoeft R. G. Nafziger E. D. Adee E. A. (2007). Nitrogen fertilization and cropping systems effects on soil organic carbon and total nitrogen pools under chisel–plow tillage in Illinois. Soil Till. Res. 95, 348–356. doi: 10.1016/j.still.2007.02.006

[B16] Jiang D. Dai T. Jing Q. Cao W. Zhou Q. Zhao H.. (2004). Effects of long–term fertilization on leaf photosynthetic characteristics and grain yield in winter wheat. Photosynthetica 42, 439–446. doi: 10.1023/B:PHOT.0000046164.77410.ef

[B17] Kautz T. Wirth S. Ellmer F. (2004). Microbial activity in a sandy arable soil is governed by the fertilization regime. Eur. J. Soil Biol. 40, 87–94. doi: 10.1016/j.ejsobi.2004.10.001

[B18] Khan A. Najeeb U. Wang L. Tan D. K. Y. Yang G. Munsif F.. (2017a). Planting density and sowing date strongly influence growth and lint yield of cotton crops. Field Crops Res. 209, 129–135. doi: 10.1016/j.fcr.2017.04.019

[B19] Khan A. Tan D. K. Y. Munsif F. Afridi M. Z. Shah F. Wei F.. (2017b). Nitrogen nutrition in cotton and control strategies for greenhouse gas emissions: a review. Environ. Sci. pollut. Res. 24, 23471–23487. doi: 10.1007/s11356-017-0131-y 28940131

[B20] Li Y. P. Gu X. B. Li Y. N. Fang P. Chen P. P. (2023). Ridge-furrow mulching combined with appropriate nitrogen rate for enhancing photosynthetic efficiency, yield and water use efficiency of summer maize in a semi-arid region of China. Agric. Water Manage. 287, 108450. doi: 10.1016/j.agwat.2023.108450

[B21] Li J. F. He J. F. Chen F. W. Tan J. H. Wu Q. X. Wan P. (2019). Status of cotton planting and fertilization research in China—Based on CNKI data analysis. China Cott. 46, 17–24,28. doi: 10.11963/1000-632X.ljfljf.20190402

[B22] Li L. L. Ma Z. B. Zhang D. L. Du Y. F. Fang W. P. Xie D. Y. (2006). Effects of applying potassium fertilizer at peak bolling stage on cotton photosynthetic characteristics and its yield and quality under different population. Plant Nutr. Fert. Sci. 12, 662–666. doi: 10.3321/j.issn:1008-505X.2006.05.010

[B23] Liang J. P. Xue Z. Q. Yang Z. Chai Z. Niu J. P. Shi Z. Y. (2021). Effects of microbial organic fertilizers onAstragalus membranaceus growth andrhizosphere microbial community. Ann. Microbiol. 71, 11. doi: 10.1186/s13213-021-01623-x

[B24] Lichtenthaler H. K. Wellburn A. R. (1983). Determinations of total carotenoids and chlorophylls a and b of leaf extracts in different solvents. Biochem. Soc Trans. 11, 591–592. doi: 10.1042/bst0110591

[B25] Liu X. Xu G. C. Wang Q. S. Hang Y. H. (2017). Effects of insect-proof net cultivation, rice-duck farming, and organic matter return on rice dry matter accumulation and nitrogen utilization. Front. Plant Sci. 8. doi: 10.3389/fpls.2017.00047 PMC5258751 28174589

[B26] Lou S. W. Dong H. Z. Tian X. L. Tian L. W. (2021). The “ Short, dense and early” Cultivation of cotton in Xinjiang: history, current situation and prospect. Sci. Agric. Sin. 54, 720–732. doi: 10.3864/j.issn.0578-1752.2021.04.005

[B27] Luo C. W. Wang R. S. Li C. N. Zheng C. H. Dou X. Y. (2023). Photosynthetic characteristics, soil nutrients, and their interspecific competitions in an apple–soybean alley cropping system subjected to different drip fertilizer regimes on the Loess Plateau, China. Agric. Water Manage. 275, 108001. doi: 10.1016/j.agwat.2022.108001

[B28] Maintang Sudding F. Asri M. Rauf A. W. (2021). Application of liquid organic and inorganic fertilizer on growth and production of hybrid maize. IOP Conf. Ser.: Earth Environ. Sci. 648, 12140. doi: 10.1088/1755-1315/648/1/012140

[B29] Mooy L. M. Hasan A. Onsili R. (2019). Growth and yield of Tomato (Lycopersicum esculantum Mill.) as influenced by the combination of liquid organic fertilizer concentration and branch pruning. IOP Conf. Ser.: Earth Environ. Sci. 260, 12170. doi: 10.1088/1755-1315/260/1/012170

[B30] Mousavi H. Cottis T. Hoff G. Solberg S. I. (2022). Nitrogen enriched organic fertilizer (neo) and its effect on ryegrass yield and soil fauna feeding activity under controlled conditions. Sustainability 14, 1–0. doi: 10.3390/su14042005

[B31] Narayan J. A. Chakravarthi M. Nerkar G. Manoj V. M. Dharshini S. Subramonian N.. (2020). Overexpression of expansin EaEXPA1, a cell wall loosening protein enhances drought tolerance in sugarcane. Ind. Crops Products 159, 113035. doi: 10.1016/j.indcrop.2020.113035

[B32] National Bureau of Statistics (2020). China Statistical Yearbook (Beijing: China Statistics Press). Available at: http://www.stats.gov.cn/xxgk/sjfb/zxfb2020/202012/t20201221_1810301.html.

[B33] Pan D. (2014). The impact of agricultural extension on farmer nutrient management behavior in Chinese rice production: a household–level analysis. Sustainability–Basel 6, 6644–6665. doi: 10.3390/su6106644

[B34] Puteri A. R. Setyowati N. Fahrurrozi F. Muktamar Z. (2021). Growth and yield of sweet corn (Zea mays L. Saccharata) as affected by inCubation time of preparation for tithonia (Tithonia diversifolia) enriched liquid organic fertilizer. IOP Conf. Ser.: Earth Environ. Sci. 637, 12090. doi: 10.1088/1755-1315/637/1/012090

[B35] Raiesi F. (2021). The quantity and quality of soil organic matter and humic substances following dry-farming and subsequent restoration in an upland pasture. Catena 202, 105249. doi: 10.1016/j.catena.2021.105249

[B36] Rodríguez A. A. Vilas J. M. Sartore G. D. Bezus R. Colazo J. Maiale S. J. (2023). Field and genetic evidence support the photosynthetic performance index (PI(ABS)) as an indicator of rice grain yield. Plant Physiol. Biochem. 201, 107897. doi: 10.1016/j.plaphy.2023.107897 37487369

[B37] Roelcke M. Han Y. Schleef K. H. Zhu J. Liu G. Cai Z.. (2004). Recent trends and recommendations for nitrogen fertilization in intensive agriculture in Eastern China. Pedosphere 14, 449–460. Available at: http://pedosphere.issas.ac.cn/trqen/ch/reader/view_abstract.aspx?file_no=200404005.

[B38] Saikia S. Talukdar M. H. Nath D (2015). Effect of organic manure application on soil microbial and biochemical properties on gerbera (Gerbera jamesonii Bolus.) varieties under naturally ventilated greenhouse. Res Crop 16, 551–554. doi: 10.5958/2348-7542.2015.00078.9

[B39] Santos E. F. D. Zanchim B. J. Campos A. G. D. Garrone R. F. Junior J. L. (2013). Photosynthesis rate, chlorophyll content and initial development of physic nut without micronutrient fertilization. Rev. Bras. Ciênc. Solo. 37, 1334–1342. doi: 10.1590/S0100-06832013000500022

[B40] Schreiber U. Bilger W. Neubauer C. (1995). Chlorophyll fluorescence as a nonintrusive indicator for rapidassessment of in *vivo* photosynthesis. Ecol. Stud. 100, 49–70. doi: 10.1007/978-3-642-79354-7_3

[B41] Sepehri A. Sanavy S. A. M. M. (2003). Water and nitrogen stress on maize photosynthesis. J. Biol. Sci. 3, 578–584. doi: 10.3923/jbs.2003.578.584

[B42] Shah A. N. Wu Y. Tanveer M. Hafeez A. Tung S. A. Ali S.. (2021). Interactive effect of nitrogen fertilizer and plant density on photosynthetic and agronomical traits of cotton at different growth stages. Saudi J. Biol. Sci. 28, 3578–3584. doi: 10.1016/j.sjbs.2021.03.034 34121901 PMC8176129

[B43] Shi X. R. Ren B. B. Jiang L. L. Fan S. X. Cao Y. L. Ma D. R. (2021). Effects of organic manure partial substitution for chemical fertilizer on the photosynthetic rate, nitrogen use efficiency and yield of rice. Chin. J. Appl. Ecol. 32, 154–162. doi: 10.13287/j.1001-9332.202101.021 33477223

[B44] Sims J. R. Haby. V. A. Haby V. A. (1971). Simplified colorimetric determination of soil organic matter. Soil Sci. 112, 137–141. doi: 10.1097/00010694-197108000-00007

[B45] Sinsawat V. Leipner J. Stamp P. Fracheboud Y. (2004). Effect of heat stress on the photosynthetic apparatus in maize (Zea mays L.) grown at control or high temperature. Environ. Exp. Bot. 52, 123–129. doi: 10.1016/j.envexpbot.2004.01.010

[B46] STATISTA Leading cotton producing countries worldwide in 2021/2022. Available at: https://www.statista.com/statistics/263055/cotton-production-worldwide-by-top-countries/.

[B47] Tian X. M. Li J. H. Wang C. Chu G. X. Wei C. Z. Zheng Q.. (2014). Effects of continuous application of bio-organic fertilizer for three years on soil nutrients, microbial biomass and enzyme activity. Soils 46, 481–488. doi: 10.13758/j.cnki.tr.2014.03.016

[B48] Tissue D. T. Thomas R. B. Strain B. R. (2010). Long–term effects of elevated CO_2_ and nutrients on photosynthesis and rubisco in loblolly pine seedlings. Plant Cell Environ. 16, 859–865. doi: 10.1111/j.1365-3040.1993.tb00508.x

[B49] Urra J. Alkorta I. Mijangos I. Garbisu C. (2020). Commercial and farm fermented liquid organic amendments to improve soil quality and lettuce yield. J. Environ. Manage. 264, 110422. doi: 10.1016/j.jenvman.2020.110422 32217314

[B50] Wang L. Xu J. Nian J. Q. Shen N. W. Lai K. K. Hu J.. (2016). Characterization and fine mapping of the rice gene OsARVL4 regulating leaf morphology and leaf vein development. Plant Growth Regul. 78, 345–356. doi: 10.1007/s10725-015-0097-z

[B51] Wang X. L. Yan Y. Xu C. C. Wang X. Y. Luo N. Dan W.. (2021). Mitigating heat impacts in maize (Zea mays L.) during the reproductive stage through biochar soil amendment. Agr. Ecosyst. Environ. 311, 107321. doi: 10.1016/j.agee.2021.107321

[B52] Wang X. G. Zhao X. H. Jiang C. J. Li C. H. Cong S. Wu D.. (2015). Effects of potassium deficiency on photosynthesis and photoprotection mechanisms in soybean (Glycine max (L.) Merr.). J. Integr. Agr. 14, 856–863. doi: 10.1016/S2095-3119(14)60848-0

[B53] Wright A. L. Hons F. M. Rouquette F. M. Jr. (2004). Long–term management impacts on soil carbon and nitrogen dynamics of grazed Bermudagrass pastures. Soil Biol. Biochem. 36, 1809–1816. doi: 10.1016/j.soilbio.2004.05.004

[B54] Wu A. Hammer G. L. Doherty A. Caemmerer S. V. C. V. Farquhar G. D. (2019). Quantifying impacts of enhancing photosynthesis on crop yield. Nat. Plants. 5, 380–388. doi: 10.1038/s41477-019-0398-8 30962528

[B55] Xiao Y. Zhang L. F. Wang N. (2017). Analysis and development of organic fertilizer industry in domestic and foreign. Agr. Eng. Tech. 37, 78–79. doi: 10.16815/j.cnki.11-5436/s.2017.26.064

[B56] Yan D. Z. Wang D. J. Yang L. Z. (2007). Long–term effect of chemical fertilizer, straw, and manure on labile organic matter fractions in a paddy soil. Biol. Fert. Soils. 44, 93–101. doi: 10.1007/s00374-007-0183-0

[B57] Yao Y. D. Liu M. Zhao H. Qin J. H. (2016). Analysis of factors affecting cotton yield in 2015 in Shihezi reclamation area, Xinjiang. China Cott. 43, 34–36. doi: 10.11963/issn.1000-632X.201605011

[B58] Yoder N. Davis J. G. (2020). Organic fertilizer comparison on growth and nutrient content of three kale cultivars. Hort. Technol. 30, 1–9. doi: 10.21273/HORTTECH04483-19

[B59] Zhang Y. P. Chen Y. Y. Yang S. J. (2013). Effects of organic and inorganic compound fertilizer application on growth and chlorophyll fluorescence characteristics in melon plants. Plant Physiol. J. 49, 722–728. doi: 10.13592/j.cnki.ppj.2013.08.002

[B60] Zhen Y. L. Li Y. B. Chen C. Ma Y. X. Chen Y. Chen D. H. (2020). Construction of optimum number of fruiting nodes benefit high yield in cotton population. Ind Crop Prod 158, 113020. doi: 10.1016/j.indcrop.2020.113020

